# Identification of a Five-Pseudogene Signature for Predicting Survival and Its ceRNA Network in Glioma

**DOI:** 10.3389/fonc.2019.01059

**Published:** 2019-10-15

**Authors:** Yulin Wang, Xin Liu, Gefei Guan, Zhe Xiao, Weijiang Zhao, Minghua Zhuang

**Affiliations:** ^1^Department of Neurosurgery, The First Affiliated Hospital of Shantou University Medical College, Shantou, China; ^2^Department of Stomatology, The First Affiliated Hospital of Shantou University Medical College, Shantou, China; ^3^Department of Neurosurgery, The First Hospital of China Medical University, Shenyang, China; ^4^Wuxi Medical College, Jiangnan University, Wuxi, China; ^5^Center for Neuroscience, Shantou University Medical College, Shantou, China

**Keywords:** glioma, pseudogene, risk signature, nomogram, ceRNA

## Abstract

**Background:** Glioma is the most common primary brain tumor with a dismal prognosis. It is urgent to develop novel molecular biomarkers and conform to individualized schemes.

**Methods:** Differentially expressed pseudogenes between low grade glioma (LGG) and glioblastoma multiforme (GBM) were identified in the training cohort. Least absolute shrinkage and selection operator (LASSO) regression and multivariate Cox proportional hazards regression analyses were used to select pseudogenes associated with prognosis of glioma. A risk signature was constructed based on the selected pseudogenes for predicting the survival of glioma patients. A pseudogene-miRNA-mRNA regulatory network was established and visualized using Cytoscape 3.5.1. Gene Oncology (GO) and signaling pathway analyses were performed on the targeted genes to investigate functional roles of the risk signature.

**Results:** Five pseudogenes (ANXA2P2, EEF1A1P9, FER1L4, HILS1, and RAET1K) correlating with glioma survival were selected and used to establish a risk signature. Time-dependent receiver operating characteristic (ROC) curves revealed that the risk signature could accurately predict the 1, 3, and 5-year survival of glioma patients. GO and signaling pathway analyses showed that the risk signature was involved in regulation of proliferation, migration, angiogenesis, and apoptosis in glioma.

**Conclusions:** In this study, a risk signature with five pseudogenes was constructed and shown to accurately predict 1-, 3-, and 5-year survival for glioma patient. The risk signature may serve as a potential target against glioma.

## Introduction

Glioma is the most common primary brain tumor and has a dismal prognosis, among which glioblastoma multiforme (GBM, WHO grade IV) is the most aggressive type with a median survival of 12–18 months and a 5 year-survival of 5% (1, 2). The standard treatment for glioma patients involves maximal surgical resection, followed by radiotherapy and chemotherapy with drugs such as temozolomide (TMZ). Unfortunately, the efficacy of these treatments is limited and the prognosis of glioma patients is still poor. TMZ resistance occurs in some patients during treatment, which is attributed to certain intrinsic and extrinsic factors, such as tumor microenvironment, heterogeneity of glioma, glioma stem cells and the sensitivity of glioma cells to chemotherapeutic drugs (3, 4). In addition, some low-grade gliomas can evolve into secondary GBM after undergoing surgical resection, radiotherapy, or chemotherapy (5). Therefore, the treatment for glioma is very complicated, and novel molecular biomarkers should be identified to conform to individualized schemes.

With the development of high-throughput sequencing technologies, non-coding RNAs have been discovered and proven to be involved in multiple cellular programs as well as many pathological processes, such as cancer (6). Increasing evidence suggests that non-coding RNAs can serve as biomarkers and therapeutic targets in cancer (7). Pseudogenes were previously considered as “genomic fossils,” but recent studies have confirmed their involvement in various biological functions (8). Some pseudogene RNAs, belonging to long non-coding RNA (lncRNA) with more than 200 nucleotides in length, act as RNA sponges for miRNAs and regulate gene expression via competing endogenous RNA (ceRNA) networks (8, 9). For example, the pseudogene PTENP1 regulate the function of PTEN through decoying PTEN-related miRNAs and competing for these miRNAs (10). In breast cancer, upregulation of PTENP1 expression can inhibit proliferation, metastasis and tumorigenicity of breast cancer cells, and enhance chemosensitivity through functioning as a sponge for miRNA-20a (11). Several studies have also demonstrated that pseudogene transcripts have histological specificity and contribute to tumorigenesis. It has been reported that 440 pseudogenes are transcribed in breast cancer, and 309 of them are differentially expressed in different breast cancer subtypes (8). The functions of pseudogenes in glioma have also been reported. PTENP1 is found to be downregulated in glioma tissues, causing inhibition of both proliferation and invasion of glioma cells (12). The annexin A2 pseudogenes (ANXA2P1, ANXA2P2, and ANXA2P3) are significantly upregulated in glioma and are associated with adverse outcome of glioma patients (13).

In this study, we identified five pseudogenes in the TCGA dataset and constructed a risk signature based on the five pseudogenes for predicting survival of glioma patients. Additionally, a nomogram was established integrating the risk signature and clinical features (age and glioma grade). Time-dependent receiver operating characteristic (ROC) curves and calibration curves were used to evaluate its efficiency and indicated a good performance for predicting 1-, 3-, and 5-year survival of glioma patients. Furthermore, competing endogenous RNA (ceRNA) regulatory networks consisting of 3 pseudogenes (ANXA2P2, EEF1A1P9, and FER1L4), their binding microRNAs (miRNAs) and target genes were established. Finally, we investigated the biological functions and pathways related to the risk signature to provide novel strategies for glioma treatment.

## Materials and Methods

### Data Set for the Study

Pseudogenes were downloaded from the HUGO Gene Nomenclature Committee (HGNC, https://www.genenames.org/). Expression data of pseudogenes and miRNA target genes as well as glioma patient clinicopathology and survival data in The Cancer Genome Atlas (TCGA) were downloaded from GlioVis (http://gliovis.bioinfo.cnio.es/) GBMLGG (RNA-seq) platform (14). In the GlioVis platform, RNA-seq data processing is based on the normalized count reads from the pre-processed data (sequence alignment and transcript abundance estimation) with log2 transformation after adding a 0.5 pseudocount. Samples in the TCGA database with detailed information of age, gender, WHO grade, survival time, and status were enrolled and were randomly divided into a training cohort (accounting for 70%) and validation cohort (accounting for 30%) using the “caret” package in R language (15). The training cohort was used to select pseudogenes and establish a prognostic risk signature in glioma, and the validation cohort was used for internal validation.

### Differentially Expressed Pseudogene Profiles

Principal component analysis (PCA) was used to assess expression distribution of available pseudogenes between low grade glioma [LGG, World Health Organization (WHO) grade II and III] (16) and glioblastoma (GBM) in the TCGA database. Differentially expressed pseudogenes were generated using R language (limma package, R version 3.5.2) between LGG and GBM samples in the training cohort. Pseudogenes with | log2(fold-change) | ≥ 2 and false discovery rate (FDR) < 0.05 were considered as differentially expressed pseudogenes.

### Identification of a Prognostic Pseudogene Signature

Univariate Cox models were used to assess the association between pseudogenes and glioma patient's overall survival (OS) from data in the training cohort. *P* < 0.05 was considered statistically significant. Least absolute shrinkage and selection operator (LASSO) regression was performed to filter the pseudogenes which were significant in univariate Cox analysis. LASSO regression can filter and select appropriate variables according to the best value of lambda, so as to eliminate over-fitting problems in the risk signature (17, 18). Subsequently, multivariate Cox regression analysis was used to further select pseudogenes by a step function in R programming language. The multivariate Cox regression analysis results were visualized in a forest plot. A risk signature was established according to the regression coefficient-weighted pseudogene expression and a risk score formula was constructed as follows (19):

$$\begin{array}{l} risk\;score=\overset{N}{\underset{i=1}{\sum }}\left(Exp_{i}*Coe_{i}\right) \end{array}$$

In the formula, *N* is the number of selected pseudogenes, *Exp*_*i*_ is the expression value of each pseudogene, and *Coe*_*i*_ is the multivariate Cox regression coefficient. Glioma patients were divided into low-risk and high-risk groups according to the median risk score value, and the performance of the prognostic risk signature was measured by Kaplan-Meier and ROC curve analyses. To better predict the 1-, 3-, and 5-year survival of glioma patients, the risk signature and several clinicopathological factors were incorporated, and a nomogram was established based on the results of the multivariate analysis by using the rms package in R language. The ROC curve and calibration curve were used to assess the efficiency of the nomogram.

### Construction of the Pseudogene-miRNA-mRNA Regulatory Network

miRNAs binding to pseudogenes were identified using the dreamBase database (http://rna.sysu.edu.cn/dreamBase/index.php) (20). miRNA target genes with at least one strong experimental method (reporter assay or western blot) were extracted from miRTarBase (http://mirtarbase.mbc.nctu.edu.tw/php/index.php) (21). Pearson analysis was performed to calculate expression correlation between pseudogenes and miRNA target genes. Target genes with | r | ≥ 0.4 were selected and Cytoscape 3.5.1 was used to construct pseudogene-miRNA-mRNA regulatory networks.

### Bioinformatics Analysis

Target genes with | r | ≥ 0.4 in the TCGA dataset (GBMLGG platform) were selected for functional analysis. Gene Oncology (GO) and KEGG pathway analyses were performed to analyze the selected target genes via the DAVID website (https://david.ncifcrf.gov/) (22). GO terms with FDR <0.001 and KEGG pathways with *P* < 0.001 were selected for charting. To validate the biological processes and signaling pathways, Metascape (http://metascape.org/gp/index.html#/main/step1) was adopted (23). Bar chart, bubble chart, and correlation heatmaps were mapped using R programming language.

### Statistical Analysis

Quantitative data were presented as the mean ± standard deviation. Statistical differences between two groups were examined by the Wilcoxon test. All statistical tests were conducted using R programming language. *P* < 0.05 was considered statistically significant.

## Results

### Five Pseudogenes Were Identified and Used for Constructing a Risk Signature for Glioma

**Figure 1:**
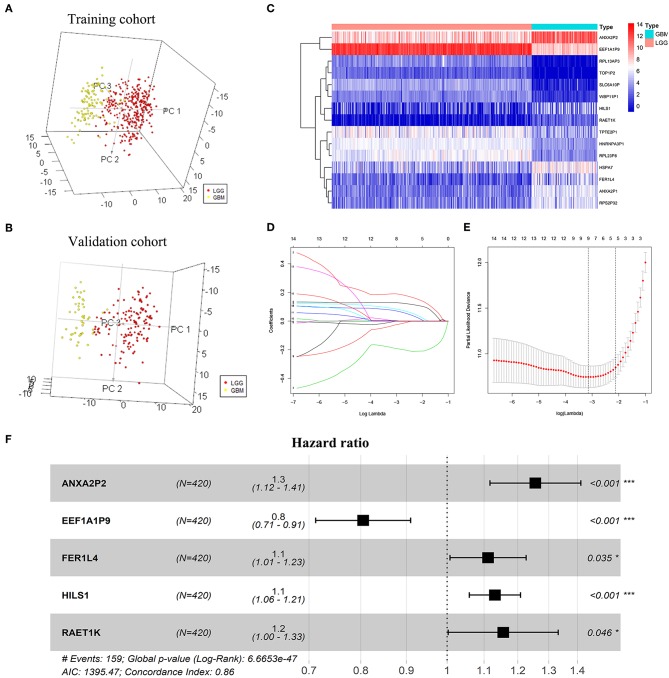
Screening pseudogenes used for constructing the risk signature for glioma. Principal components analysis of pseudogenes between glioblastoma (GBM) and low grade glioma (LGG) in the training cohort **(A)** and the validation cohort **(B)**. **(C)** Heatmap showed the pseudogenes differentially expressed between LGG and GBM in the training cohort (| log2(fold-change) | ≥ 2 and FDR < 0.05). **(D)** Log (Lambda) value of the 15 pseudogenes in LASSO model. **(E)** The most appropriate log (Lambda) value in the LASSO model. **(F)** Multivariate Cox regression analysis was performed and five pseudogenes (ANXA2P2, EEF1A1P9, FER1L4, HILS1, and RAET1K) were selected to construct the risk signature.

A total of 263 pseudogenes were included in the TCGA dataset. Glioma patients in the TCGA dataset (GBMLGG) with detailed clinical information (gender, age, WHO grade, survival time, and status) were enrolled and randomly divided into training cohort (*n* = 420) and validation cohort (*n* = 178) (Table 1). PCA was performed to explore the distribution of these pseudogenes between LGG and GBM samples in the training cohort and the validation cohort, respectively. In the training cohort, GBM is distributed on the left side, while LGG is gathered on the other side (Figure 1A). Similar result was also observed in the validation cohort (Figure 1B), indicating different distributions and functional roles of the pseudogenes in LGG and GBM. Considering the grade dependence in glioma, we chose differentially expressed pseudogenes between LGG and GBM for further study, which can reflect the distinct characteristics of LGG and GBM. Fifteen differentially expressed pseudogenes were identified in the training cohort, including seven upregulated pseudogenes and eight downregulated pseudogenes in GBM (Figure 1C). The 15 pseudogenes were all correlated with glioma prognosis according to univariate Cox analysis (Supplementary Table 1, *P* < 0.0001). To select appropriate parameters for constructing a risk signature, LASSO regression was used and 9 pseudogenes were identified (ANXA2P2, EEF1A1P9, FER1L4, HILS1, HSPA7, RAET1K, RPL13AP3, RPS2P32, and TPTE2P1) (Figures 1D,E). Finally, multivariate Cox regression analysis was performed on the nine pseudogenes, and five pseudogenes (ANXA2P2, EEF1A1P9, FER1L4, HILS1, and RAET1K) were retained using the “step” function (Figure 1E). Information about the five pseudogenes was obtained through the dreamBase database and is listed in Table 2. EEF1A1P9 was a protective factor (HR < 1), and ANXA2P2, FER1L4, HILS1, and RAET1K were defined as risk factors (HR > 1) in glioma (Figure 1F).

**Table 1:** Clinicopathological characteristics of samples in the training cohort and the validation cohort.

**Clinicopathological characteristics**	**No. of samples**	
	**Training cohort** **(*n* = 420)**	**Validation cohort** **(*n* = 178)**
**Pathological Type**		
Astrocytoma	121	46
Oligodendroglioma	195	87
GBM	104	45
**Who Grade**		
Grade II	148	63
Grade III	168	70
Grade IV	104	45
**Age, Years**		
Average value	48	47
Range	14–89	18–87
**Gender**		
Male	234	114
Female	186	64

**Table 2:** Information on the five pseudogenes identified by dreamBase.

**Pseudogene**	**Ensembl ID**	**Genome location**	**Gene type**
ANXA2P2	ENST00000435128	chr9:33624274-33625293	processed_pseudogene
EEF1A1P9	ENST00000514975	chr4:105484698-105486080	processed_pseudogene
FER1L4	ENST00000615531	chr20:35558866-35607494	transcribed_unitary_pseudogene
HILS1	ENST00000545329	chr17:50171514-50171936	transcribed_unitary_pseudogene
RAET1K	ENST00000403651	chr6:150000090-150005157	transcribed_unprocessed_pseudogene

### The Five Pseudogenes Were Prognostic Biomarkers in Glioma

**Figure 2:**
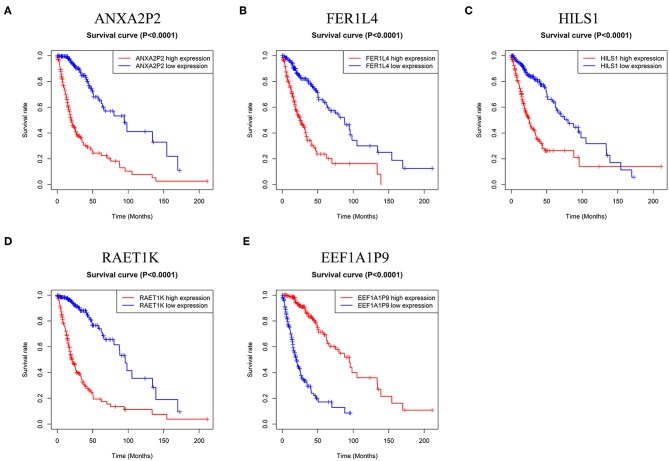
Kaplan-Meier survival curves for the five pseudogenes in glioma. **(A–D)** High expression level of ANXA2P2, FER1L4, HILS1, and RAET1K indicated poorer prognosis in glioma patients (*P* < 0.0001). **(E)** Glioma patients with higher expression level of EEF1A1P9 had favorable prognosis (*P* < 0.0001).

To confirm the prognostic value of the five pseudogenes, we performed Kaplan-Meier analysis in the training cohort. As shown in Figure 2, higher expression of ANXA2P2, FER1L4, HILS1, or RAET1K resulted in poorer patient prognosis than that in the lower expression group (Figures 2A–D, *P* < 0.0001). On the contrary, patients with higher expression of EEF1A1P9 had favorable prognosis (Figure 2E, *P* < 0.0001). Combined with multivariate Cox regression analysis, these results suggest the potential values of the five pseudogenes as prognostic biomarkers for glioma patients.

### Construction of the Prognostic Risk Signature With Five Pseudogenes in Glioma

**Figure 3:**
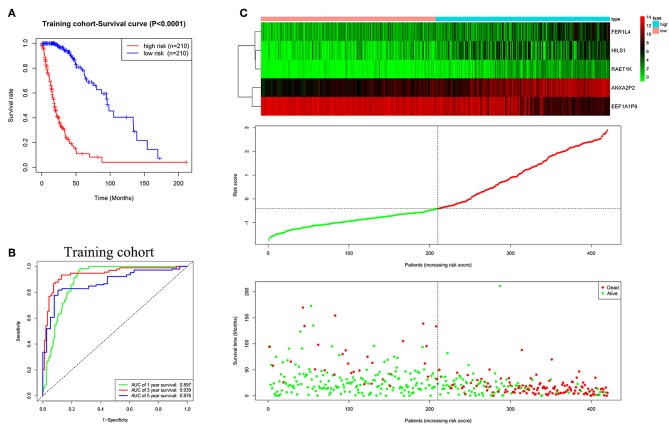
Characteristics of the five-pseudogene risk signature in the training cohort. **(A)** Survival curves for high-risk and low-risk groups classified by the risk signature in the training cohort. **(B)** ROC curves for the 1-, 3-, and 5-year survival according to the five-pseudogene risk signature in the training cohort. **(C)** Glioma expression profiles of the five pseudogenes, risk score distributions and patient survival in the training cohort.

Based on multivariate Cox regression analysis, the five pseudogenes (ANXA2P2, EEF1A1P9, FER1L4, HILS1, and RAET1K) were integrated to establish a risk signature in the training cohort. The risk scores were calculated using the formula mentioned in the methods, as follows: risk score = (0.2279 × expression level of ANXA2P2) + (−0.2170 × expression level of EEF1A1P9) + (0.1056 × expression level of FER1L4) + (0.1232 × expression level of HILS1) + (0.1448 × expression level of RAET1K). Patients in the training cohort were divided into low-risk (*n* = 210) and high-risk (*n* = 210) groups according to the median risk score. The K-M curve showed poorer prognosis in the high-risk group than low-risk group (Figure 3A, *P* < 0.0001). The ROC curve was used to evaluate the efficacy to predict 1-, 3-, and 5-year survival in glioma patients. The areas under curve (AUC) for 1-, 3-, and 5-year survival were 0.897, 0.939, and 0.876, respectively (Figure 3B). As shown in Figure 3C, with the increase of risk scores, the expression level of EEF1A1P9 decreased, whereas the expression levels of ANXA2P2, FER1L4, HILS1, and RAET1K were upregulated. Meanwhile, the number of patient deaths increased.

### Validation of the Prognostic Five-Pseudogene Risk Signature

**Figure 4:**
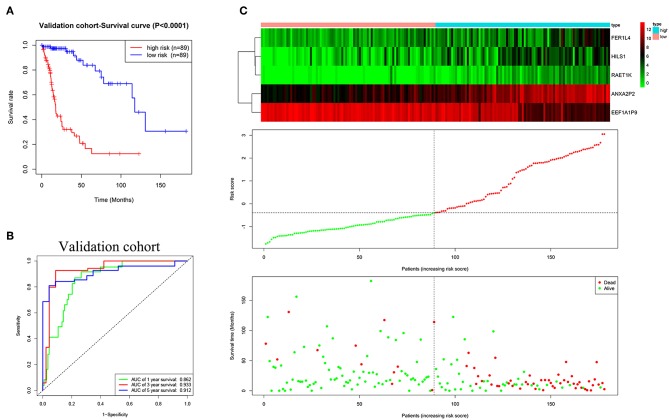
Evaluating the efficacy of the five pseudogenes risk signature in the validation cohort. **(A)** Survival curves for high-risk and low-risk groups classified by the risk signature in the validation cohort. **(B)** ROC curves for the 1-, 3-, and 5-year survival according to the five-pseudogene risk signature in the validation cohort. **(C)** Glioma expression profiles of the five pseudogenes, risk score distributions and patient survival in the validation cohort.

The validation cohort was applied to assess the performance of the five-pseudogene risk signature. Glioma patients in the validation cohort were divided into low-risk (*n* = 89) and high-risk (*n* = 89) groups according to the median risk score. The K-M curve showed patients in the low-risk group had a favorable prognosis (Figure 4A, *P* < 0.0001). AUCs for 1-, 3-, and 5-year survival were 0.862, 0.933, and 0.912, respectively (Figure 4B). In agreement with the results in the training cohort, the expression level of EEF1A1P9 were downregulated and other genes were upregulated with an increase in risk scores; correspondingly, the number of patient deaths increased (Figure 4C).

### Construction of a Nomogram Integrating the Risk Signature and Clinicopathological Factors

**Figure 5:**
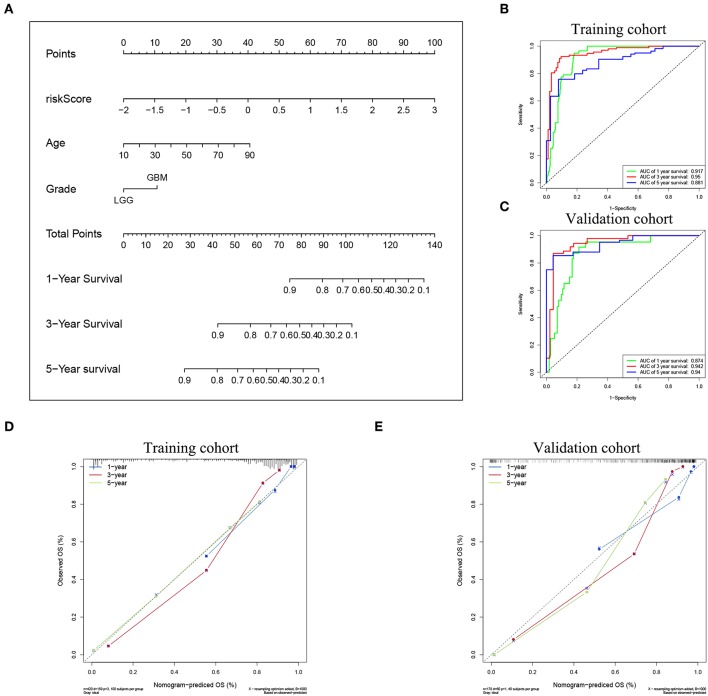
Nomogram for predicting the survival rate of glioma patient. **(A)** A nomogram was established based on the risk signature, age, and grade for predicting survival of glioma patient. ROC curves were used for evaluating the efficiency of the nomogram. **(B)** AUCs for predicting 1-, 3-, and 5-year survival were 0.917, 0.95, and 0.881, respectively, in the training cohort. **(C)** AUCs for predicting 1-, 3-, and 5-year survival were 0.874, 0.942, and 0.94, respectively, in the validation cohort. Calibration plot of observed and predicted probabilities for the nomogram in the training cohort **(D)** and validation cohort **(E)**.

To confirm the prognostic value of the risk signature, univariate and multivariate Cox regression analysis were conducted in the training cohort, and showed that the risk signature with five pseudogenes was independently associated with overall survival of glioma (Table 3). Considering clinical relevance and prognostic value of age and grade, a nomogram based on the risk signature, age and glioma grade was established in the training cohort. The validation cohort was used to assess its efficiency. As the nomogram shows, the incidences of 1-, 3-, and 5-year survival can be estimated according to the point total, which is the sum of the points in each item (Figure 5A). The AUCs for the 1-, 3-, and 5-year incidences of survival in the training cohort were 0.917, 0.95, and 0.881, respectively (Figure 5B). In the validation cohort, the AUCs of the 1-, 3-, and 5-year incidences of survival were 0.874, 0.942, and 0.94, respectively (Figure 5C). Calibration curves for estimating 1-, 3-, and 5-year survival showed that there were good correlations between the prediction and observation in both the training cohort (Figure 5D) and validation cohort (Figure 5E). These results suggest that the nomogram can accurately predict 1-, 3-, and 5-year survival of glioma patients.

**Table 3:** Univariate and multivariate analysis of the risk signature and clinical prognostic factors in the training cohort.

**Variable**	**Univariate analysis**		**Multivariate analysis**	
	**HR**	***P*-value**	**HR**	***P*-value**
Risk score	2.7181	<0.0001	2.7780	<0.0001
Age	1.0663	<0.0001	1.0263	0.0003
**Grade**				
LGG	Reference		Reference	
GBM	8.8504	<0.0001	1.7329	0.1001
**Gender**			–	–
Female	Reference			
Male	1.1086	0.5227		

### Associations Between the Risk Signature and Clinicopathologic Features in Glioma

**Figure 6:**
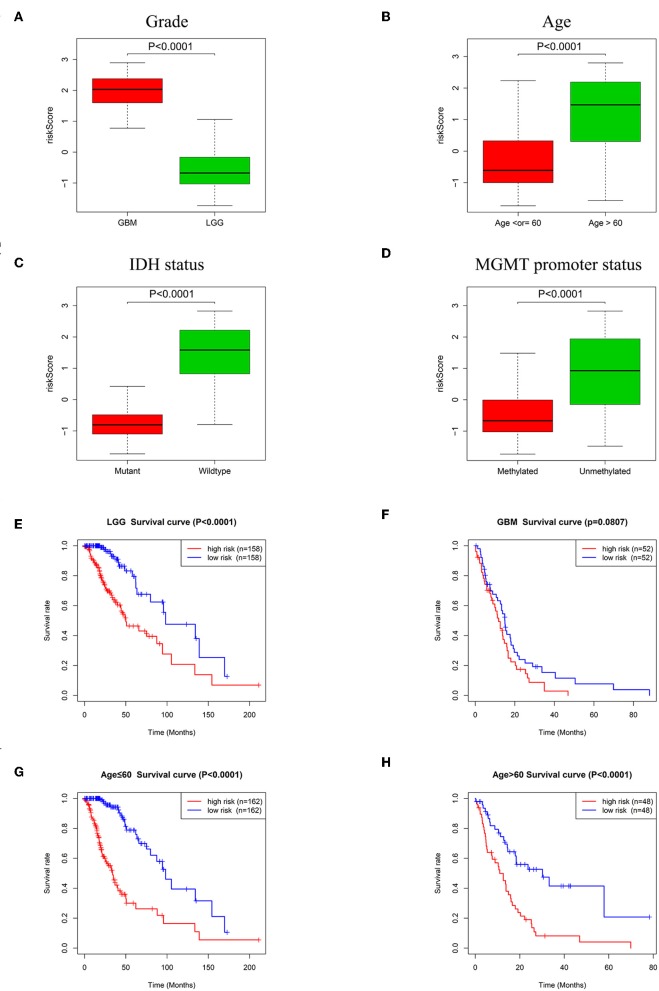
Association between the risk signature and different cohorts stratified by glioma grade, age, IDH status and MGMT promoter status. **(A)** Risk scores in GBM were higher than that in LGG (*P* < 0.0001). **(B)** Patients >60 years old had higher risk scores than patients ≤60 years old (*P* < 0.0001). **(C)** Risk scores in IDH mutation samples were lower than IDH wildtype samples (*P* < 0.0001). **(D)** Risk scores in MGMT promoter methylated samples decreased compared with samples with the MGMT promoter unmethylated (*P* < 0.0001). **(E)** K-M survival curves indicated the high-risk group had adverse outcome in LGG (*P* < 0.0001). **(F)** K-M survival curve for the high-risk group and low-risk group in GBM (*P* = 0.0807). Patients in the high-risk group had poorer prognosis than patients in the low-risk group independent of age ≤60 (**G**, *P* < 0.0001) or age >60 (**H**, *P* < 0.0001).

To explore the relationships between the risk signature and clinicopathologic characteristics, we investigated the levels of risk score in different cohorts stratified by glioma grade, age, IDH status, and MGMT promoter status. In different grades, GBM (WHO grade IV) had higher risk scores than LGG (Figure 6A, *P* < 0.0001). Age is a risk factor for glioma patient survival. Glioma patients were therefore divided into two groups according to age (>60 and ≤60). As shown in Figure 6B, the risk scores of patients >60 of age were much higher than patients with age ≤60. For IDH status, risk scores decreased in patients with an IDH mutant compared with patients with wildtype IDH (Figure 6C, *P* < 0.0001). The risk scores of patients with a methylated MGMT promoter were lower than patients without MGMT promoter methylation (Figure 6D, *P* < 0.0001).

The relationships between the risk signature and glioma patient survival in LGG, GBM and groups stratified by age (>60 and ≤60) were also explored. Kaplan-Meier curves showed that the patients with high risk scores had poorer prognosis than patients with low risk scores in LGG (Figure 6E, *P* < 0.0001). Although there was no statistical difference between high-risk group and low-risk group in GBM, the tendency of the two curves was obvious (Figure 6F, *P* = 0.0807). In regard to groups stratified by age, the survival time of patients with low risk scores were longer than the patients with high risk scores whether in the ≤60 years old age group (Figure 6G, *P* < 0.0001) or in the group with age >60 (Figure 6H, *P* < 0.0001).

### Pseudogene-miRNA-mRNA Regulatory Networks

**Figure 7:**
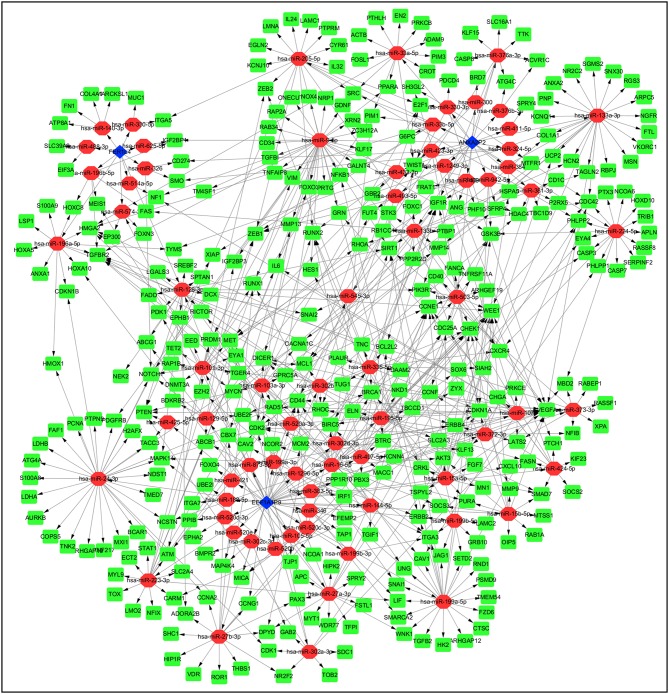
Construction of pseudogene-miRNA-mRNA regulatory networks. Pseudogenes together with binding miRNAs and target genes related to the three pseudogenes with | r | ≥ 0.4 were used to construct the pseudogene-miRNA-mRNA regulatory networks. Blue diamonds represented pseudogenes, which are located at the cores of the networks. Red ellipses and green rectangles stand for binding miRNAs and target genes, respectively.

Pseudogenes can positively or negatively regulate gene expression by functioning as miRNA decoys. Thus, pseudogene-expressed RNA is also known as competing endogenous RNA (ceRNA) (24). Potential miRNAs binding to the six pseudogenes were identified using the dreamBase database and are listed in Supplementary Table 2. miRNA target genes with at least one strong experimental method (reporter assay or western blot) were extracted from miRTarBase database. Pearson analysis was used to calculate expression correlation between each pseudogene and its miRNA target genes. Target genes with | r | ≥ 0.4 were picked up and listed in Supplementary Table 3. Ultimately, three pseudogenes (ANXA2P2, EEF1A1P9, and FER1L4), together with 72 microRNAs and 322 targeted genes were used to construct the pseudogene-miRNA-mRNA regulatory networks and visualized using Cytoscape (Figure 7).

### The Pseudogenes (ANXA2P2, FER1L4, and EEF1A1P9) Were Highly Correlated With Important Regulatory Genes in Glioma

**Figure 8:**
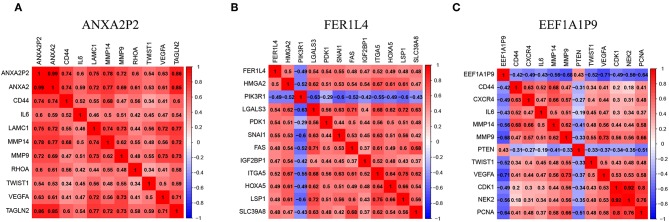
Pseudogenes (ANXA2P2, FER1L4, and EEF1A1P9)-related genes in glioma. **(A–C)** The heatmaps showed some representative genes highly correlated with the three pseudogenes (| r | ≥ 0.4).

To better present the correlation between pseudogenes and their miRNA target genes, correlation heatmaps were conducted. ANXA2P2 was positively correlated with genes that are related to glioma proliferation, invasion and angiogenesis, such as ANXA2, CD44, IL6, MMP14, MMP9, and VEGFA (Figure 8A). FER1L4 was positively correlated with SNAIL1, IGF2BP1, and HOXA5 (Figure 8B). On the contrary, EEF1A1P9 was negatively correlated with CD44, IL6, MMP9, MMP14, VEGFA, NEK2, and PCNA, but positively correlated with PTEN (Figure 8C). These results indicate the three pseudogenes might play important roles in glioma occurrence and progression.

### Functional Analysis of the Five-Pseudogene Risk Signature

**Figure 9:**
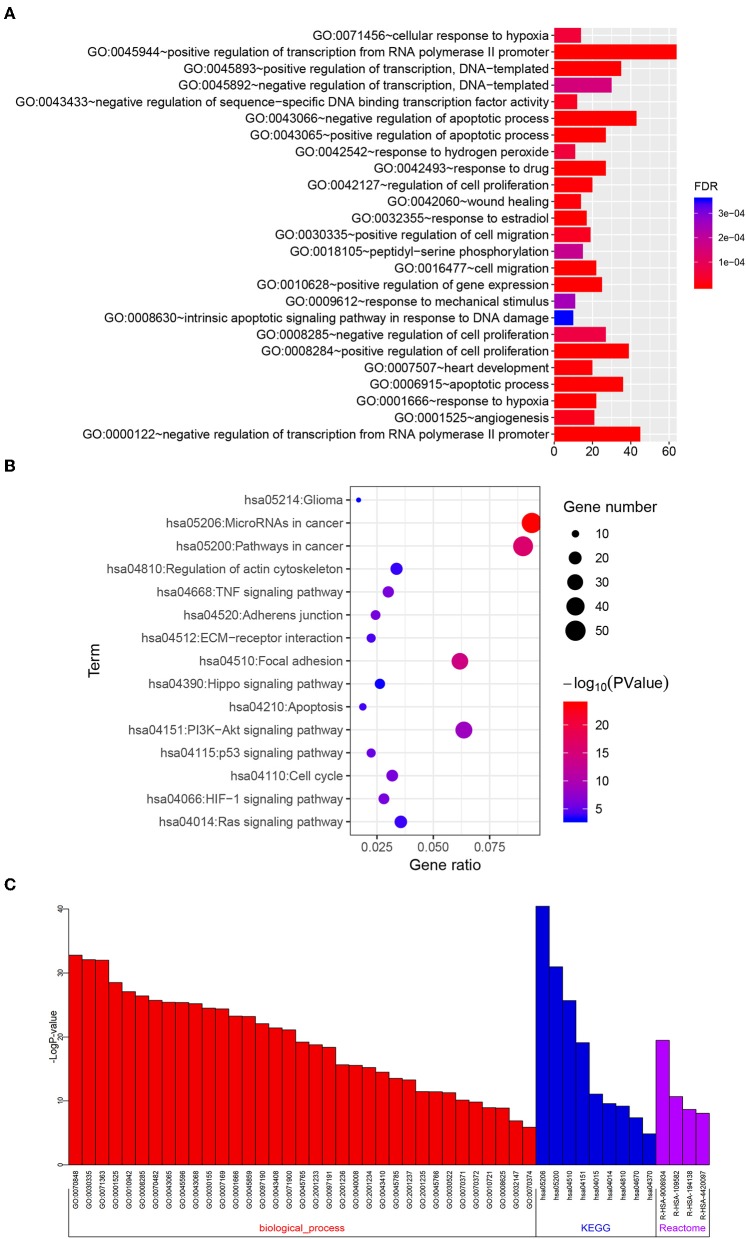
Functional roles of the six-pseudogene risk signature. Gene oncology **(A)** and KEGG pathway **(B)** analyses were performed on the related target genes via DAVID. **(C)** Metascape was used to confirm the functional and pathway analysis in biological processes, KEGG, and Reactome pathways.

To investigate the functional roles of the risk signature, related genes with | r | ≥ 0.4 were used for GO and KEGG pathway analyses via DAVID. We discovered that the signature was functionally associated with biological processes that are related to tumor apoptosis, proliferation, migration, and angiogenesis, such as regulation of cell proliferation, cell migration, apoptosis, response to hypoxia, and angiogenesis (Figure 9A, FDR < 0.001). Correspondingly, several KEGG pathways including focal adhesion, apoptosis, the PI3K-AKT signaling pathway, cell cycle, HIF-1 signaling pathway and ECM receptor interaction were identified (Figure 9B, *P* < 0.001). To further confirm these results, Metascape was performed on the related genes. As shown in Figure 9C and Supplementary Table 4, similar terms such as angiogenesis, response to hypoxia, regulation of cell adhesion, focal adhesion and PI3K-Akt signaling pathway were also obtained. Collectively, these results indicate the risk signature is correlated with the function of proliferation, migration, angiogenesis, and apoptosis in glioma.

## Discussion

Glioma is one of the most aggressive brain tumors without effective treatment. Current research shows that lncRNAs are extensively involved in glioma formation and progression, and can serve as promising therapeutic targets (25). As a special group of lncRNAs, pseudogenes are remnants of their parental genes that lost their ability to encode proteins (9). It has been reported, based on supervised analysis, that 71 pseudogenes were found differentially expressed among GBM subtypes (classical, mesenchymal, neural, and proneural), indicating their potential roles in glioma (26). In our study, we screened out five pseudogenes (ANXA2P2, EEF1A1P9, FER1L4, HILS1, and RAET1K) that were differentially expressed between LGG and GBM and were associated with the prognosis of glioma patients. Among the five pseudogenes, ANXA2P2 is a pseudogene of ANXA2 and is located on chromosomes 9 (13). The expression level of ANXA2 is positively related to ANXA2P2 expression, and both are significantly upregulated in diffuse glioma, as well as mesenchymal subtype of GBM. Moreover, high expression level of ANXA2P2 is associated with adverse outcome of glioma patients (13). Our findings are consistent with this and indicate ANXA2P2 can be a candidate therapeutic target for glioma. FER1L4 is a pseudogene located on chromosome 20 (27, 28), and it is downregulated in gastric cancer, lung cancer and hepatocellular carcinoma tissues (28–30). It also has been reported that FER1L4 can inhibit proliferation and metastasis in lung cancer and hepatocellular carcinoma via regulating the PI3K/AKT signal pathway and enhance chemosensitivity of ovarian cancer through the MAPK signal pathway (29–31). However, a recent study shows that FER1L4 is upregulated in glioma and promotes glioma proliferation and tumorigenicity (32). In support of this, our findings also suggest that higher expression of FER1L4 predicts unfavorable prognosis in glioma patients. For the remaining three pseudogenes (EEF1A1P9, HILS1, and RAET1K), there are few reports on their biological functions at present. In our study, we show that EEF1A1P9 has an HR < 1, indicating it is a protective factor in glioma, and higher expression level of EEF1A1P9 is associated with better prognosis. On the contrary, higher expression levels of HILS1 and RAET1K indicate an adverse outcome in glioma. Thus, the three pseudogenes might be used as prognostic biomarkers in glioma, and future studies will be performed to explore their biological functions.

Accumulating evidence suggests vital roles for pseudogenes in multiple cellular processes and various cancers. Mechanistically, some pseudogenes with specific miRNA target sites are capable of regulating gene expression via acting as ceRNAs (10). In this study, we constructed the pseudogene-miRNA-mRNA regulatory networks to show the relationships of the pseudogenes (ANXA2P2, FER1L4, and EEF1A1P9) along with their binding miRNAs and target genes. The three pseudogenes highly correlate with expression levels of several target genes, such as IL6, MMP14, MMP9, VEGFA, and SNAIL1 that participate in glioma development (33–35). To explore the biological functions and signaling pathways of the risk signature, GO and pathway analysis were performed on these target genes, many terms associated with glioma progression were obtained, such as regulation of cell proliferation, cell migration, apoptotic process, response to hypoxia, angiogenesis, focal adhesion, the PI3K-AKT signaling pathway, cell cycle, and HIF-1 signaling pathway.

A prior report that constructed another signature with six pseudogenes (SP3P, ANXA2P3, PTTG3P, LPAL2, CLCA3P, and TDH) for prediction of glioma patient survival (36). However, our study made some innovations and also showed better performance for predicting survival of glioma patients. Firstly, LASSO regression was used to identify pseudogenes, so as to avoid over-fitting problems. Secondly, we constructed the pseudogene-miRNA-mRNA regulatory networks and uncovered the functional roles of the risk signature in glioma based on the highly correlated genes. Thirdly, we established a nomogram based on the risk signature and clinicopathological factors (age and grade) that more accurately could predict the 1-, 3-, and 5-year survival for glioma patients. Even with the above advantages, there were some problems remaining. Firstly, the data were downloaded from the TCGA database with limited numbers of patients. Secondly, the nomogram was established based on the five pseudogenes, with some clinical factors of glioma not taken into account, such as surgical resection, radiotherapy and chemotherapy. Thirdly, internal validation was used to evaluate the efficiency of the risk signature rather than external validation, because of unavailability to get integrated data of the five pseudogenes from other databases, such as the REMBRANDT and Gravendeel datasets. In future studies, we may collect more glioma samples and detailed clinical information to validate the five-pseudogene signature.

In conclusion, we identified five pseudogenes associated with glioma patient survival. By combining the five pseudogenes, a risk signature was established and validated to be competent to predict the 1-, 3-, and 5-year survival of glioma patients. The risk signature was correlated with glioma grade, age, IDH, and MGMT promoter status. Furthermore, the pseudogene-miRNA-mRNA regulatory networks were constructed. In light of the targeted genes, GO and KEGG pathway analyses showed that the risk signature might be involved in regulating biological processes and signaling pathways related to glioma progression. Taken together, the risk signature in our study may serve as a prognostic biomarker for glioma.

## Data Availability Statement

All data generated or analyzed during this study are included in this published article and its Supplementary Information Files.

## Author Contributions

YW, XL, and GG conceived the concept and implemented the scheme. YW and WZ wrote the original draft. ZX and MZ reviewed and edited the manuscript. All authors read and approved the final manuscript.

### Conflict of Interest

The authors declare that the research was conducted in the absence of any commercial or financial relationships that could be construed as a potential conflict of interest.
