# Supplementary material for: Identification of a Five-Pseudogene Signature for Predicting Survival and Its ceRNA Network in Glioma
Source: Front Oncol. 2019 Oct 15;9:1059. doi: 10.3389/fonc.2019.01059 (PMC6803554; doi:10.3389/fonc.2019.01059)
Supplement: Supplementary file 1 [file Table_1.DOCX]

**Supplementary Table 1. Prognostic value of the 15 differentially expressed pseudogenes using univariate Cox analysis**

| Pseudogene | HR | z | P value |
| --- | --- | --- | --- |
| ANXA2P1 | 1.777589 | 12.75289 | 3.00E-37 |
| ANXA2P2 | 1.737478 | 12.23972 | 1.91E-34 |
| EEF1A1P9 | 0.590701 | -13.3383 | 1.39E-40 |
| FER1L4 | 1.533979 | 10.77764 | 4.39E-27 |
| HILS1 | 1.300539 | 8.746802 | 2.19E-18 |
| HNRNPA3P1 | 0.532625 | -11.8566 | 1.99E-32 |
| HSPA7 | 1.529756 | 10.06887 | 7.58E-24 |
| RAET1K | 1.872699 | 11.80232 | 3.80E-32 |
| RPL13AP3 | 0.566308 | -12.4147 | 2.17E-35 |
| RPL23P8 | 0.560778 | -12.6399 | 1.27E-36 |
| RPS2P32 | 1.751233 | 11.27381 | 1.77E-29 |
| SLC6A10P | 0.650409 | -11.2691 | 1.86E-29 |
| TOP1P2 | 0.525544 | -10.6999 | 1.02E-26 |
| TPTE2P1 | 0.67474 | -10.2035 | 1.91E-24 |
| WBP11P1 | 0.560057 | -10.4161 | 2.09E-25 |
