# Supplementary material for: Identification of a Five-Pseudogene Signature for Predicting Survival and Its ceRNA Network in Glioma
Source: Front Oncol. 2019 Oct 15;9:1059. doi: 10.3389/fonc.2019.01059 (PMC6803554; doi:10.3389/fonc.2019.01059)
Supplement: Supplementary file 2 [file Table_2.DOCX]

**Supplementary table 2. Potential microRNAs binding to the 5 pseudogenes identified by dreamBase.**

| **pseudogene** | **miRNA** |
| --- | --- |
| ANXA2P2 | hsa-miR-1249-3p; hsa-miR-1287-5p; hsa-miR-1301-3p; hsa-miR-133a-3p; hsa-miR-133b; hsa-miR-1343-3p; hsa-miR-205-5p; hsa-miR-224-5p; hsa-miR-3167; hsa-miR-324-5p;  hsa-miR-330-3p; hsa-miR-33a-5p; hsa-miR-33b-5p; hsa-miR-3614-5p; hsa-miR-376a-3p;  hsa-miR-376b-3p; hsa-miR-380-3p; hsa-miR-384; hsa-miR-409-3p; hsa-miR-411-5p;  hsa-miR-423-3p; hsa-miR-431-5p; hsa-miR-433-3p; hsa-miR-493-5p; hsa-miR-503-5p;  hsa-miR-5047; hsa-miR-512-3p; hsa-miR-516b-5p; hsa-miR-552-5p; hsa-miR-579-5p;  hsa-miR-6746-5p; hsa-miR-6783-3p; hsa-miR-876-5p; hsa-miR-892c-5p; hsa-miR-942-5p; hsa-miR-9-5p |
| EEF1A1P9 | hsa-miR-101-3p; hsa-miR-103a-3p; hsa-miR-105-5p; hsa-miR-107; hsa-miR-1193;  hsa-miR-1270; hsa-miR-128-3p; hsa-miR-129-5p; hsa-miR-1296-5p; hsa-miR-1343-3p;  hsa-miR-144-5p; hsa-miR-15a-5p; hsa-miR-15b-5p; hsa-miR-16-5p; hsa-miR-188-5p;  hsa-miR-195-5p; hsa-miR-199a-3p; hsa-miR-199a-5p; hsa-miR-199b-3p; hsa-miR-199b-5p; hsa-miR-2114-3p; hsa-miR-216a-3p; hsa-miR-223-3p; hsa-miR-2355-5p; hsa-miR-24-3p;  hsa-miR-2681-5p; hsa-miR-27a-3p; hsa-miR-27b-3p; hsa-miR-302a-3p; hsa-miR-302b-3p; hsa-miR-302c-3p ; hsa-miR-302d-3p; hsa-miR-302e; hsa-miR-3129-5p; hsa-miR-3164;  hsa-miR-3200-3p; hsa-miR-323b-3p; hsa-miR-335-5p; hsa-miR-346; hsa-miR-3614-5p;  hsa-miR-362-5p; hsa-miR-3681-3p; hsa-miR-372-3p; hsa-miR-373-3p; hsa-miR-383-5p;  hsa-miR-421; hsa-miR-424-5p; hsa-miR-425-5p; hsa-miR-432-5p; hsa-miR-497-5p;  hsa-miR-499b-5p; hsa-miR-500b-5p; hsa-miR-503-5p; hsa-miR-512-3p; hsa-miR-520a-3p; hsa-miR-520b; hsa-miR-520c-3p; hsa-miR-520d-3p; hsa-miR-520e; hsa-miR-545-3p;  hsa-miR-551a; hsa-miR-551b-3p; hsa-miR-574-3p; hsa-miR-620; hsa-miR-624-5p;  hsa-miR-627-5p; hsa-miR-628-5p; hsa-miR-642a-3p; hsa-miR-642b-3p; hsa-miR-6509-3p; hsa-miR-670-3p; hsa-miR-6783-3p; hsa-miR-6820-3p; hsa-miR-6823-3p; hsa-miR-6838-5p; hsa-miR-6866-3p; hsa-miR-7853-5p; hsa-miR-873-5p; hsa-miR-888-5p |
| FER1L4 | hsa-miR-299-3p; hsa-miR-3173-5p; hsa-miR-3180; hsa-miR-3180-3p; hsa-miR-3196;  hsa-miR-326; hsa-miR-330-5p; hsa-miR-488-3p; hsa-miR-514a-5p; hsa-miR-526b-5p;  hsa-miR-542-3p; hsa-miR-556-5p; hsa-miR-625-5p; hsa-miR-670-3p; hsa-miR-6799-3p;  hsa-miR-6816-5p; hsa-miR-874-3p |
| RAET1K | hsa-miR-1245b-5p; hsa-miR-3142 |
| HILS1 | 0 |
