# Supplementary material for: Identification of a Five-Pseudogene Signature for Predicting Survival and Its ceRNA Network in Glioma
Source: Front Oncol. 2019 Oct 15;9:1059. doi: 10.3389/fonc.2019.01059 (PMC6803554; doi:10.3389/fonc.2019.01059)
Supplement: Supplementary file 3 [file Table_3.DOCX]

**Supplementary table 3. miRNA targeted genes correlated with their pseudogenes at | r |≥ 0.4.**

| **pseudogene** | **miRNA targeted genes** |
| --- | --- |
| **ANXA2P2** | HDAC4 RAP2A PHLPP1 SIRT1 PIK3R1 NF1 SH3GL2 PHLPP2 RB1CC1 BRD7 KCNJ10 PTCH1 CBX7 IGF1R PPP2R2D BCL2L2 FRAT1 P2RX5 FOXO3 PRTG ZEB1 HCN2 PDCD4 ZEB2 ACVR1C PRKCB GDNF SRC KLF15 PTPRM GSK3B SNX30 PPARA NOTCH1 ONECUT2 CACNA1C G6PC RBPJ NCOA6 PHF10 TBC1D9 XIAP NR2C2 CDKN1A CD34 IL24 FANCA RASSF8 CD1C CCNE1 ERBB2 TNFRSF11A MCL1 ATG4C FOXC1 HES1 MMP13 PDGFRB MET IL32 PTHLH EGLN2 TTK TRIB1 PIM3 FUT4 EN2 NGFR CCNG1 SLC16A1 SERPINF2 ARHGEF19 CHEK1 IGF2BP1 SMO RUNX2 PRDM1 KCNQ1 SPRY4 EYA4 APLN TYMS CD274 PTBP1 SNAI2 TGFBR2 TWIST1 XRN2 CYR61 UCP2 NFKB1 PIM1 STK3 RGS3 PNP KLF17 IL6 RHOA HOXD10 GALNT4 NRP1 VEGFA MTFR1 ZC3H12A CASP3 ADAM9 LMNA TM4SF1 CXCR4 CROT GBP2 NOX4 GRN HMGA2 SFRP4 CASP7 FAS WEE1 IGF2BP3 SGMS2 CDK2 TGFBI HSPA5 CDC42 FTL CD40 MMP9 VKORC1 COL1A1 CD44 RUNX1 ACTB ARPC5 LAMC1 FOSL1 RHOC PTX3 MMP14 RAB34 MBD2 CASP8 TNFAIP8 ANG VIM ITGA5 MSN TAGLN2 ANXA2 |
| **EEF1A1P9** | LDHA VEGFA IGF2BP3 MMP9 EFEMP2 FSTL1 DPYD LGALS3 PDK1 PLAUR RAP1B PCNA SHC1 CASP3 UBE2F VDR ECT2 ZYX HMGA2 WEE1 PAX3 CXCL10 TGIF1 SIAH2 WDR77 CCNA2 CDK2 MBD2 LIF MCM2 SDC1 MACC1 KIF23 COL1A1 CDC42 LATS2 MMP14 NEK2 SOCS3 UBE2I SNAI2 FADD OIP5 SOCS2 GPRC5A CARM1 RAD51 VIM ATG4A HSPA5 SPRY2 THBS1 BIRC5 ITGA7 BDKRB2 HMOX1 HOXA10 CCNE1 RAB1A CAV2 TWIST1 CCNF PTPN9 FANCA RHOC FZD6 TACC3 IRF1 TBCCD1 CD40 UNG S100A8 TGFB2 SNAI1 CDK1 CXCR4 RUNX1 SFRP4 MYL9 ZNF217 TMEM54 ROR1 CTSC ITGA3 TNC AURKB CHEK1 RASSF1 E2F1 MMP13 ELN PPIB EYA4 SLC2A3 NCSTN MET GRN RHOA PSMD9 EPHA2 MICA JAG1 FAF1 MEIS1 EED H2AFX TMED7 KCNN4 CAV1 TFPI GRB10 IL6 DNMT3A PTGER4 STAT1 COPS5 BRCA1 TAP1 CD44 ARHGEF19 LMO2 PBX3 ADORA2B NR2F2 PDGFRB EZH2 MAPK14 CDC25A HK2 LAMC2 ARHGAP19 RND1 DCX SETD2 NOTCH1 TOB2 APC MYCN NCOR2 NFIX SMARCA2 ABCG1 CACNA1C NFIB XPA CHGA PTEN ABCB1 TSPYL2 PRKCE MECP2 PHLPP2 EYA1 NDST1 WNK1 BCAR1 AKT3 TJP1 TOX ERBB4 CDKN1B EP300 MTSS1 ZEB2 TET2 FGF7 BCL2L2 DICER1 TUG1 FOXN3 CRKL SLC2A4 PPP1R10 DAAM2 EPHB1 NCOA1 BMPR2 GAB2 PIK3R1 CBX7 FOXO3 SREBF2 MYT1 PTCH1 ATM SH3GL2 RABEP1 KLF13 PURA LDHB MAP4K4 BTRC FASN ZEB1 HIP1R SOX6 NKD1 ARHGAP12 NF1 IGF1R FOXO4 TNK2 PHLPP1 MN1 SMAD7 SPTAN1 HIPK2 MXI1 SIRT1 HDAC4 RICTOR |
| **FER1L4** | EIF3A SIRT1 NF1 PIK3R1 RICTOR EPHB1 ATP8A1 SREBF2 SPTAN1 MARCKSL1 WEE1 SNAI2 HOXC8 S100A9 FN1 MUC1 IGF2BP1 ANXA1 COL4A1 FAS LSP1 SLC39A8 HOXA5 HMGA2 ITGA5 PDK1 LGALS3 SNAI1 |
| **RAET1K** | 0 |
| **HILS1** | 0 |
