# Supplementary material for: Identification of a Five-Pseudogene Signature for Predicting Survival and Its ceRNA Network in Glioma
Source: Front Oncol. 2019 Oct 15;9:1059. doi: 10.3389/fonc.2019.01059 (PMC6803554; doi:10.3389/fonc.2019.01059)
Supplement: Supplementary file 4 [file Table_4.DOCX]

**Supplementary table 4. Gene Oncology and pathways analysis via Metascape.**

| **Category** | **Term** | **Description** | **LogP** |
| --- | --- | --- | --- |
| GO Biological Processes | GO:0001568 | blood vessel development | -34.5523 |
| GO Biological Processes | GO:0070848 | response to growth factor | -32.78726 |
| GO Biological Processes | GO:0048514 | blood vessel morphogenesis | -32.71667 |
| GO Biological Processes | GO:0030335 | positive regulation of cell migration | -32.09283 |
| GO Biological Processes | GO:2000147 | positive regulation of cell motility | -32.08214 |
| GO Biological Processes | GO:0071363 | cellular response to growth factor stimulus | -32.00099 |
| GO Biological Processes | GO:0040017 | positive regulation of locomotion | -31.84884 |
| GO Biological Processes | GO:0051272 | positive regulation of cellular component movement | -31.5075 |
| GO Biological Processes | GO:0001525 | angiogenesis | -28.50194 |
| GO Biological Processes | GO:0010942 | positive regulation of cell death | -27.09171 |
| GO Biological Processes | GO:0008285 | negative regulation of cell proliferation | -26.42131 |
| GO Biological Processes | GO:0002009 | morphogenesis of an epithelium | -26.14643 |
| GO Biological Processes | GO:0048729 | tissue morphogenesis | -26.04617 |
| GO Biological Processes | GO:0070482 | response to oxygen levels | -25.73981 |
| GO Biological Processes | GO:0007507 | heart development | -25.42702 |
| GO Biological Processes | GO:0043065 | positive regulation of apoptotic process | -25.41541 |
| GO Biological Processes | GO:0045596 | negative regulation of cell differentiation | -25.38198 |
| GO Biological Processes | GO:0043068 | positive regulation of programmed cell death | -25.1957 |
| GO Biological Processes | GO:0030855 | epithelial cell differentiation | -24.53035 |
| GO Biological Processes | GO:0030155 | regulation of cell adhesion | -24.52303 |
| GO Biological Processes | GO:0007169 | transmembrane receptor protein tyrosine kinase signaling pathway | -24.37682 |
| GO Biological Processes | GO:0048732 | gland development | -23.92134 |
| GO Biological Processes | GO:0050673 | epithelial cell proliferation | -23.92134 |
| GO Biological Processes | GO:0071407 | cellular response to organic cyclic compound | -23.79324 |
| GO Biological Processes | GO:0042060 | wound healing | -23.28715 |
| GO Biological Processes | GO:0001666 | response to hypoxia | -23.26146 |
| GO Biological Processes | GO:0045859 | regulation of protein kinase activity | -23.22073 |
| GO Biological Processes | GO:0009611 | response to wounding | -23.08025 |
| GO Biological Processes | GO:0036293 | response to decreased oxygen levels | -22.77892 |
| GO Biological Processes | GO:0048608 | reproductive structure development | -22.35966 |
| GO Biological Processes | GO:0061458 | reproductive system development | -22.24458 |
| GO Biological Processes | GO:0097190 | apoptotic signaling pathway | -22.07526 |
| GO Biological Processes | GO:0043408 | regulation of MAPK cascade | -21.42766 |
| GO Biological Processes | GO:0071900 | regulation of protein serine/threonine kinase activity | -21.10922 |
| GO Biological Processes | GO:0001501 | skeletal system development | -21.04395 |
| GO Biological Processes | GO:0061061 | muscle structure development | -20.17876 |
| GO Biological Processes | GO:2000027 | regulation of animal organ morphogenesis | -20.11534 |
| GO Biological Processes | GO:0032870 | cellular response to hormone stimulus | -20.08568 |
| GO Biological Processes | GO:0050678 | regulation of epithelial cell proliferation | -19.43901 |
| GO Biological Processes | GO:0001667 | ameboidal-type cell migration | -19.27707 |
| GO Biological Processes | GO:0045765 | regulation of angiogenesis | -19.21422 |
| GO Biological Processes | GO:0071396 | cellular response to lipid | -19.14641 |
| GO Biological Processes | GO:2001233 | regulation of apoptotic signaling pathway | -18.77686 |
| GO Biological Processes | GO:1901342 | regulation of vasculature development | -18.63582 |
| GO Biological Processes | GO:0010632 | regulation of epithelial cell migration | -18.4464 |
| GO Biological Processes | GO:0097191 | extrinsic apoptotic signaling pathway | -18.38453 |
| GO Biological Processes | GO:0010631 | epithelial cell migration | -17.88166 |
| GO Biological Processes | GO:0090132 | epithelium migration | -17.76845 |
| GO Biological Processes | GO:0048589 | developmental growth | -17.76522 |
| GO Biological Processes | GO:0090130 | tissue migration | -17.54546 |
| GO Biological Processes | GO:0048545 | response to steroid hormone | -17.28222 |
| GO Biological Processes | GO:1901699 | cellular response to nitrogen compound | -16.85524 |
| GO Biological Processes | GO:0071417 | cellular response to organonitrogen compound | -16.80575 |
| GO Biological Processes | GO:0009792 | embryo development ending in birth or egg hatching | -16.37768 |
| GO Biological Processes | GO:0048568 | embryonic organ development | -16.20412 |
| GO Biological Processes | GO:0010634 | positive regulation of epithelial cell migration | -16.11161 |
| GO Biological Processes | GO:0043009 | chordate embryonic development | -16.09671 |
| GO Biological Processes | GO:0003006 | developmental process involved in reproduction | -16.02529 |
| GO Biological Processes | GO:2001236 | regulation of extrinsic apoptotic signaling pathway | -15.6519 |
| GO Biological Processes | GO:0040008 | regulation of growth | -15.57517 |
| GO Biological Processes | GO:0060562 | epithelial tube morphogenesis | -15.48137 |
| GO Biological Processes | GO:2001234 | negative regulation of apoptotic signaling pathway | -15.23393 |
| GO Biological Processes | GO:0048598 | embryonic morphogenesis | -15.21925 |
| GO Biological Processes | GO:1901652 | response to peptide | -14.64652 |
| GO Biological Processes | GO:0001763 | morphogenesis of a branching structure | -14.61601 |
| GO Biological Processes | GO:0014706 | striated muscle tissue development | -14.60423 |
| GO Biological Processes | GO:0043410 | positive regulation of MAPK cascade | -14.49215 |
| GO Biological Processes | GO:0060537 | muscle tissue development | -14.09961 |
| GO Biological Processes | GO:0007517 | muscle organ development | -14.07073 |
| GO Biological Processes | GO:0051347 | positive regulation of transferase activity | -13.65281 |
| GO Biological Processes | GO:0045785 | positive regulation of cell adhesion | -13.53529 |
| GO Biological Processes | GO:2001237 | negative regulation of extrinsic apoptotic signaling pathway | -13.28316 |
| GO Biological Processes | GO:0061138 | morphogenesis of a branching epithelium | -13.24262 |
| GO Biological Processes | GO:0071453 | cellular response to oxygen levels | -12.90689 |
| GO Biological Processes | GO:0045860 | positive regulation of protein kinase activity | -12.54531 |
| GO Biological Processes | GO:0061448 | connective tissue development | -12.45453 |
| GO Biological Processes | GO:0035265 | organ growth | -12.34288 |
| GO Biological Processes | GO:0071456 | cellular response to hypoxia | -12.09335 |
| GO Biological Processes | GO:0048638 | regulation of developmental growth | -11.88925 |
| GO Biological Processes | GO:0050878 | regulation of body fluid levels | -11.75624 |
| GO Biological Processes | GO:0048754 | branching morphogenesis of an epithelial tube | -11.72295 |
| GO Biological Processes | GO:0036294 | cellular response to decreased oxygen levels | -11.6959 |
| GO Biological Processes | GO:0033674 | positive regulation of kinase activity | -11.66127 |
| GO Biological Processes | GO:0043542 | endothelial cell migration | -11.60518 |
| GO Biological Processes | GO:0001935 | endothelial cell proliferation | -11.50638 |
| GO Biological Processes | GO:2001235 | positive regulation of apoptotic signaling pathway | -11.46781 |
| GO Biological Processes | GO:0045766 | positive regulation of angiogenesis | -11.42557 |
| GO Biological Processes | GO:0010594 | regulation of endothelial cell migration | -11.39328 |
| GO Biological Processes | GO:0110110 | positive regulation of animal organ morphogenesis | -11.36758 |
| GO Biological Processes | GO:0030522 | intracellular receptor signaling pathway | -11.28023 |
| GO Biological Processes | GO:0043434 | response to peptide hormone | -11.27474 |
| GO Biological Processes | GO:1904018 | positive regulation of vasculature development | -11.24673 |
| GO Biological Processes | GO:0043405 | regulation of MAP kinase activity | -11.16057 |
| GO Biological Processes | GO:0051216 | cartilage development | -11.15009 |
| GO Biological Processes | GO:0043281 | regulation of cysteine-type endopeptidase activity involved in apoptotic process | -11.15009 |
| GO Biological Processes | GO:1901653 | cellular response to peptide | -10.88686 |
| GO Biological Processes | GO:0045862 | positive regulation of proteolysis | -10.64991 |
| GO Biological Processes | GO:0046620 | regulation of organ growth | -10.55863 |
| GO Biological Processes | GO:0050679 | positive regulation of epithelial cell proliferation | -10.41543 |
| GO Biological Processes | GO:2000116 | regulation of cysteine-type endopeptidase activity | -10.31748 |
| GO Biological Processes | GO:0070371 | ERK1 and ERK2 cascade | -10.13574 |
| GO Biological Processes | GO:0052547 | regulation of peptidase activity | -10.12208 |
| GO Biological Processes | GO:0032868 | response to insulin | -9.931491 |
| GO Biological Processes | GO:0070372 | regulation of ERK1 and ERK2 cascade | -9.829673 |
| GO Biological Processes | GO:0071902 | positive regulation of protein serine/threonine kinase activity | -9.740491 |
| GO Biological Processes | GO:0071383 | cellular response to steroid hormone stimulus | -9.579235 |
| GO Biological Processes | GO:1902041 | regulation of extrinsic apoptotic signaling pathway via death domain receptors | -9.570679 |
| GO Biological Processes | GO:0071375 | cellular response to peptide hormone stimulus | -9.454527 |
| GO Biological Processes | GO:0001701 | in utero embryonic development | -9.439844 |
| GO Biological Processes | GO:0048738 | cardiac muscle tissue development | -9.353604 |
| GO Biological Processes | GO:0001936 | regulation of endothelial cell proliferation | -9.328078 |
| GO Biological Processes | GO:0043401 | steroid hormone mediated signaling pathway | -9.254573 |
| GO Biological Processes | GO:0032869 | cellular response to insulin stimulus | -9.138077 |
| GO Biological Processes | GO:0045927 | positive regulation of growth | -9.084061 |
| GO Biological Processes | GO:0014855 | striated muscle cell proliferation | -9.082125 |
| GO Biological Processes | GO:0010721 | negative regulation of cell development | -8.953071 |
| GO Biological Processes | GO:0008625 | extrinsic apoptotic signaling pathway via death domain receptors | -8.894188 |
| GO Biological Processes | GO:0060038 | cardiac muscle cell proliferation | -8.865568 |
| GO Biological Processes | GO:0010595 | positive regulation of endothelial cell migration | -8.845696 |
| GO Biological Processes | GO:0050768 | negative regulation of neurogenesis | -8.625732 |
| GO Biological Processes | GO:0009755 | hormone-mediated signaling pathway | -8.457669 |
| GO Biological Processes | GO:0048010 | vascular endothelial growth factor receptor signaling pathway | -8.266566 |
| GO Biological Processes | GO:0008286 | insulin receptor signaling pathway | -8.198383 |
| GO Biological Processes | GO:0007596 | blood coagulation | -8.190227 |
| GO Biological Processes | GO:0051961 | negative regulation of nervous system development | -8.151729 |
| GO Biological Processes | GO:0007599 | hemostasis | -8.07796 |
| GO Biological Processes | GO:0050817 | coagulation | -8.055757 |
| GO Biological Processes | GO:0052548 | regulation of endopeptidase activity | -7.876596 |
| GO Biological Processes | GO:0055017 | cardiac muscle tissue growth | -7.813133 |
| GO Biological Processes | GO:0016202 | regulation of striated muscle tissue development | -7.664409 |
| GO Biological Processes | GO:1901861 | regulation of muscle tissue development | -7.55751 |
| GO Biological Processes | GO:0030518 | intracellular steroid hormone receptor signaling pathway | -7.535083 |
| GO Biological Processes | GO:0048634 | regulation of muscle organ development | -7.522419 |
| GO Biological Processes | GO:0060419 | heart growth | -7.49116 |
| GO Biological Processes | GO:0043535 | regulation of blood vessel endothelial cell migration | -7.453031 |
| GO Biological Processes | GO:0010952 | positive regulation of peptidase activity | -7.342928 |
| GO Biological Processes | GO:1902043 | positive regulation of extrinsic apoptotic signaling pathway via death domain receptors | -7.223945 |
| GO Biological Processes | GO:0060043 | regulation of cardiac muscle cell proliferation | -7.134682 |
| GO Biological Processes | GO:0055021 | regulation of cardiac muscle tissue growth | -7.047382 |
| GO Biological Processes | GO:0043406 | positive regulation of MAP kinase activity | -7.012914 |
| GO Biological Processes | GO:0055024 | regulation of cardiac muscle tissue development | -6.94858 |
| GO Biological Processes | GO:0032147 | activation of protein kinase activity | -6.881849 |
| GO Biological Processes | GO:0060420 | regulation of heart growth | -6.736846 |
| GO Biological Processes | GO:0043534 | blood vessel endothelial cell migration | -6.694087 |
| GO Biological Processes | GO:0007519 | skeletal muscle tissue development | -6.492648 |
| GO Biological Processes | GO:0001938 | positive regulation of endothelial cell proliferation | -6.486481 |
| GO Biological Processes | GO:0060538 | skeletal muscle organ development | -6.224431 |
| GO Biological Processes | GO:0002062 | chondrocyte differentiation | -6.146671 |
| GO Biological Processes | GO:0043280 | positive regulation of cysteine-type endopeptidase activity involved in apoptotic process | -6.005176 |
| GO Biological Processes | GO:0033143 | regulation of intracellular steroid hormone receptor signaling pathway | -5.955332 |
| GO Biological Processes | GO:0048639 | positive regulation of developmental growth | -5.892379 |
| GO Biological Processes | GO:0070374 | positive regulation of ERK1 and ERK2 cascade | -5.876064 |
| GO Biological Processes | GO:0043536 | positive regulation of blood vessel endothelial cell migration | -5.862509 |
| GO Biological Processes | GO:0045665 | negative regulation of neuron differentiation | -5.711572 |
| GO Biological Processes | GO:2001056 | positive regulation of cysteine-type endopeptidase activity | -5.45925 |
| GO Biological Processes | GO:0061035 | regulation of cartilage development | -5.413109 |
| GO Biological Processes | GO:2001238 | positive regulation of extrinsic apoptotic signaling pathway | -5.359202 |
| GO Biological Processes | GO:0060045 | positive regulation of cardiac muscle cell proliferation | -4.9106 |
| GO Biological Processes | GO:0030521 | androgen receptor signaling pathway | -4.821526 |
| GO Biological Processes | GO:0010950 | positive regulation of endopeptidase activity | -4.690426 |
| GO Biological Processes | GO:0046622 | positive regulation of organ growth | -4.634938 |
| GO Biological Processes | GO:0046626 | regulation of insulin receptor signaling pathway | -4.634938 |
| GO Biological Processes | GO:1900076 | regulation of cellular response to insulin stimulus | -4.379169 |
| GO Biological Processes | GO:0030168 | platelet activation | -4.349659 |
| GO Biological Processes | GO:0055023 | positive regulation of cardiac muscle tissue growth | -4.327298 |
| GO Biological Processes | GO:0061036 | positive regulation of cartilage development | -4.206984 |
| GO Biological Processes | GO:0060421 | positive regulation of heart growth | -4.131471 |
| KEGG Pathway | hsa05206 | MicroRNAs in cancer | -40.45139 |
| KEGG Pathway | hsa05200 | Pathways in cancer | -30.97145 |
| KEGG Pathway | hsa04510 | Focal adhesion | -25.7049 |
| KEGG Pathway | hsa05205 | Proteoglycans in cancer | -25.41468 |
| KEGG Pathway | hsa04151 | PI3K-Akt signaling pathway | -19.10285 |
| KEGG Pathway | hsa05161 | Hepatitis B | -17.64701 |
| KEGG Pathway | hsa05100 | Bacterial invasion of epithelial cells | -11.84133 |
| KEGG Pathway | hsa05222 | Small cell lung cancer | -11.21859 |
| KEGG Pathway | hsa04015 | Rap1 signaling pathway | -11.07341 |
| KEGG Pathway | hsa04014 | Ras signaling pathway | -9.57792 |
| KEGG Pathway | hsa04810 | Regulation of actin cytoskeleton | -9.204115 |
| KEGG Pathway | hsa05418 | Fluid shear stress and atherosclerosis | -8.118302 |
| KEGG Pathway | hsa04670 | Leukocyte transendothelial migration | -7.360405 |
| KEGG Pathway | hsa04370 | VEGF signaling pathway | -4.87044 |
| Reactome Gene Sets | R-HSA-9006934 | Signaling by Receptor Tyrosine Kinases | -19.47663 |
| Reactome Gene Sets | R-HSA-109582 | Hemostasis | -10.68787 |
| Reactome Gene Sets | R-HSA-194138 | Signaling by VEGF | -8.694735 |
| Reactome Gene Sets | R-HSA-4420097 | VEGFA-VEGFR2 Pathway | -8.059092 |
| Reactome Gene Sets | R-HSA-76002 | Platelet activation, signaling and aggregation | -7.74735 |
